# Comparative Gene Expression Analysis throughout the Life Cycle of *Leishmania braziliensis*: Diversity of Expression Profiles among Clinical Isolates

**DOI:** 10.1371/journal.pntd.0001021

**Published:** 2011-05-10

**Authors:** Vanessa Adaui, Denis Castillo, Mirko Zimic, Andres Gutierrez, Saskia Decuypere, Manu Vanaerschot, Simonne De Doncker, Kathy Schnorbusch, Ilse Maes, Gert Van der Auwera, Louis Maes, Alejandro Llanos-Cuentas, Jorge Arevalo, Jean-Claude Dujardin

**Affiliations:** 1 Instituto de Medicina Tropical Alexander von Humboldt, Universidad Peruana Cayetano Heredia, Lima, Peru; 2 Unit of Molecular Parasitology, Department of Parasitology, Institute of Tropical Medicine, Antwerp, Belgium; 3 Laboratorios de Investigación y Desarrollo, Facultad de Ciencias y Filosofía, Universidad Peruana Cayetano Heredia, Lima, Peru; 4 Department of Biomedical Sciences, Faculty of Pharmaceutical, Biomedical and Veterinary Sciences, University of Antwerp, Antwerp, Belgium; University of Pittsburgh, United States of America

## Abstract

**Background:**

Most of the *Leishmania* genome is reported to be constitutively expressed during the life cycle of the parasite, with a few regulated genes. Inter-species comparative transcriptomics evidenced a low number of species-specific differences related to differentially distributed genes or the differential regulation of conserved genes. It is of uppermost importance to ensure that the observed differences are indeed species-specific and not simply specific of the strains selected for representing the species. The relevance of this concern is illustrated by current study.

**Methodology/Principal Findings:**

We selected 5 clinical isolates of *L. braziliensis* characterized by their diversity of clinical and *in vitro* phenotypes. Real-time quantitative PCR was performed on promastigote and amastigote life stages to assess gene expression profiles at seven time points covering the whole life cycle. We tested 12 genes encoding proteins with roles in transport, thiol-based redox metabolism, cellular reduction, RNA poly(A)-tail metabolism, cytoskeleton function and ribosomal function. The general trend of expression profiles showed that regulation of gene expression essentially occurs around the stationary phase of promastigotes. However, the genes involved in this phenomenon appeared to vary significantly among the isolates considered.

**Conclusion/Significance:**

Our results clearly illustrate the unique character of each isolate in terms of gene expression dynamics. Results obtained on an individual strain are not necessarily representative of a given species. Therefore, extreme care should be taken when comparing the profiles of different species and extrapolating functional differences between them.

## Author Summary

*Leishmania* is a group of parasites (Protozoa, Trypanosomatidae) responsible for a wide spectrum of clinical forms. Among the factors explaining this phenotypic polymorphism, parasite features are important contributors. One approach to identify them consists in characterizing the gene expression profiles throughout the life cycle. In a recent study, the transcriptome of 3 *Leishmania* species was compared and this revealed species-specific differences, albeit in a low number. A key issue, however, is to ensure that the observed differences are indeed species-specific and not specific of the strains selected for representing the species. In order to illustrate the relevance of this concern, we analyzed here the gene expression profiles of 5 clinical isolates of *L. braziliensis* at seven time points of the life cycle. Our results clearly illustrate the unique character of each isolate in terms of gene expression dynamics: one *Leishmania* strain is not necessarily representative of a given species.

## Introduction

*Leishmania* are digenic Protozoan parasites endemic worldwide and causing a spectrum of diseases in humans collectively referred to as leishmaniasis. As part of their life cycle, *Leishmania* alternate between the alimentary tract of the sandfly vector (where they grow as extracellular flagellated promastigotes and differentiate into infective non-dividing metacyclic forms) and the phagolysosome of the vertebrate host macrophages (where parasites differentiate into aflagellated replicative amastigotes). Important morphological and biochemical changes underlie the differentiations involved in the life cycle and are most likely the result of regulated changes in gene expression in response to environmental signals (e.g. temperature change and pH shift) [1], [2], [3]. A recent study assessing gene expression profiles throughout the life cycle of a single strain of *L. infantum* showed that most of the *Leishmania* genome is constitutively expressed and that consequently, regulated genes are a minority [4]. Interestingly, most significant events of regulation were shown to occur during promastigote development, with a dramatic down-regulation during the transition from promastigotes to amastigotes, hereby supporting the hypothesis of pre-adaptation of the parasite to intracellular survival in the macrophage [4].

In parallel to these longitudinal studies of gene expression throughout the life cycle of a given strain, another critical dimension needs to be explored, i.e. the gene expression diversity within natural populations of *Leishmania*. Indeed, these parasites successfully colonized a large range of hosts in various ecological niches, hereby determining different transmission types in which humans play a variable role (from dead-end host to reservoir). Not surprisingly, the parasites are characterized by high phenotypic diversity: infectivity, tissue tropism, clinical pattern or drug susceptibility, among others. This polymorphism is reflected at the taxonomic level, with more than 20 described species. A first comparative transcriptomic study throughout the life cycle of 3 strains belonging to the species *L. major*, *L. infantum* and *L. braziliensis* was recently reported: this provided in the 3 species a similar picture of a mostly conservative gene expression, and a minority of species-specific differences related to differentially distributed genes or the differential regulation of conserved genes, either of which are subject to translational and/or post-translational controls [5]. This type of research being undertaken among other reasons for a better understanding of the differences in virulence and pathogenicity of the respective species, it is of utmost importance to ensure that the observed differences are indeed species-specific or simply specific to the strains selected as representative of the species.

In a previous study targeting a limited number of genes, we analyzed the expression profile of 21 *L. braziliensis* clinical isolates during *in vitro* promastigote growth [6]. A fraction of genes were up-regulated during differentiation, but the set of regulated genes varied between isolates, providing a picture of intra-species gene expression mosaic. We aimed here to continue our assessment of gene expression diversity within a single species (*L. braziliensis*) by extending it to the whole life cycle of the parasite. This type of study is complicated by the extreme sensitivity of the amastigote stage to disturbance of the intracellular parasite's environment during harvesting, hence extreme care is needed to ‘freeze’ gene expression levels instantly [7]. Our previous work also demonstrated the importance of time-course analyses to better observe the dynamics of gene expression changes during *in vitro* growth and to allow more reliable comparisons between different strains [6], [7]. These considerations currently impede high-throughput experiments involving the clinically relevant amastigote stage. We surveyed here 5 clinical isolates of *L. braziliensis,* characterized by their diversity of clinical and *in vitro* phenotypes. We applied a standardized and reproducible biological protocol [6], [7] and used real-time quantitative PCR to assess gene expression profiles at seven time points covering the whole life cycle. We tested 12 genes encoding proteins with roles in transport, thiol-based redox metabolism, cellular reduction, RNA poly(A)-tail metabolism and housekeeping functions.

## Materials and Methods

### Ethical statements

The five parasite isolates used here were obtained from patients with cutaneous leishmaniasis at the Instituto de Medicina Tropical A. von Humboldt, Lima, Peru. Protocol and informed consent were approved by the Research Ethic Committees of Universidad Peruana Cayetano Heredia (Lima, Peru) and Institute of Tropical Medicine (Antwerp, Belgium). Written, informed consent was obtained from all participating subjects or their legal guardians. Animal experimentation concerned only the use of peritoneal macrophages obtained from mice. Our animal protocol adhered to the guidelines at the Universidad Peruana Cayetano Heredia, Lima, Peru and was in agreement with the Peruvian and Belgian regulations for the protection and welfare of laboratory animals. Mouse care and experimental procedures were performed under approval of the Ethic Committee of the Universidad Peruana Cayetano Heredia as well as the Animal Ethic Committee of the Institute of Tropical Medicine Antwerp (PAR-018/2).

### Parasites

The five parasite isolates were obtained from Peruvian patients with confirmed cutaneous leishmaniasis and originating from 4 regions of the country (Table 1). The selected isolates were previously characterized as *L. braziliensis* by PCR-RFLP assays targeting a range of markers [8], but we retyped them here to obtain a more precise perception of their genetic diversity. Therefore, we sequenced part of the coding region of the *hsp70* genes, a locus recently shown to be phylogenetically very informative [9] and more and more used for species identification of Neotropical *Leishmania* species [10]–[12]. Sequencing concerned the isolates of this study [GenBank accession numbers: FR715986 (isolate PER002), FR715987 (isolate PER006), FR715988 (isolate PER182), FR715989 (isolate PER104), FR715990 (isolate PER163)] and the additional Peruvian *L. braziliensis* reference strain LEM2222 (http://www.parasitologie.univ-montp1.fr/cnrl.htm; GenBank accession number: FR715991). The sequences were aligned with reference strains recently published by our group [9] and available GenBank entries, and compared by Neighbor-Joining analysis of p-distances, using the software package MEGA4 (http://www.megasoftware.net/). Data on the *in vitro* Sb^V^ and Sb^III^ susceptibility of the studied isolates, as tested by the intracellular amastigote-macrophage model [8], and of the clinical treatment outcome of respective patients, were available (see Table 1 for summary of isolates' features). Isolates were used here for gene expression analysis within a maximum of 25 *in vitro* passages post-isolation from patients.

**Table 1:** Geographical origin and *in vitro* features of Peruvian *L. braziliensis* isolates included in the study.

International code	Origin	Sb^V^	Sb^III^	Tx outcome	Prom (slope)	Prom (max density)	% inf. Mf	N. ama/Mf
MHOM/PE/03/PER163/0	Huanuco, Leoncio Prado	2	0	Definite cure	1.0×10^7^	2.6×10^7^	88	3.6
MHOM/PE/01/PER002/0	Madre de Dios, Tambopata	6	2	Unresponsive	0.8×10^7^	1.6×10^7^	25	1.2
MHOM/PE/01/PER006/1	Junin, Satipo	6+	nd	Unresponsive	1.0×10^7^	2.6×10^7^	37	1.4
MHOM/PE/02/PER104/0	Madre de Dios, Tambopata	6+	6+	Unresponsive	2.0×10^7^	3.1×10^7^	85	3.3
MHOM/PE/03/PER182/0	Ayacucho, La Mar	6	5	Definite cure	2.0×10^7^	2.6×10^7^	27	1.2

**Sb^V^** and **Sb^III^**: *in vitro* susceptibility to antimony (pentavalent and trivalent, respectively), expressed as an ‘activity index’ (A.I.), i.e. as the ratio of the ED_50_ (50% effective dose) of that tested isolate to the ED_50_ of the WHO reference *L. braziliensis* strain MHOM/BR/75/M2903. Isolates with an A.I. of 0–2 were considered sensitive to the tested drug (0, more sensitive than the reference strain M2903), while isolates with an A.I. of 3 or higher were considered resistant. Data shown were reported in Yardley *et al*. [8]. **Tx outcome**: clinical treatment outcome at the 12-month follow-up of respective patients. **Prom (slope)**: the slope of the promastigote growth curve was calculated from the equation of the best-fitted line, considering all time points until maximum density was reached (from duplicate experiments). **Prom (max density)**: maximal density achieved during stationary phase; average of the densities at the time when maximal density was reached and 24 hours later (from duplicate experiments). **% inf. Mf**: percentage of infected macrophages at 24 hours post-infection. **N. ama/Mf**: average number of amastigotes per macrophage at 24 hours post-infection; all amastigote experiments were done in duplicate. **nd**: not done.

### *In vitro* promastigote production

The *in vitro* conditions for promastigote generation have been described previously [6]. Briefly, growth curves were initiated by inoculating 3×10^6^ parasites/mL in 5 mL medium 199 (M199; Sigma) containing 20% heat-inactivated fetal bovine serum (FBS; Lonza Bioscience), 25 mM Hepes (pH 7.4), 100 units/mL penicillin and 100 µg/mL streptomycin (Lonza). Two independently grown cultures and corresponding harvests at 24 h (early-log phase), 72 h (late-log phase), 120 h (early-stationary phase) and 168 h (late-stationary phase) time points were performed in parallel for each isolate (biological replicates).

### *In vitro* intracellular amastigote production

Intracellular amastigotes were obtained after infection with axenic amastigote-like forms, as this was reported to enhance infectivity in species of *L. (Viannia)* subgenus [13]. Briefly, stationary-phase promastigotes were incubated in M199-20% FBS, at pH 5.5 and 34°C, during 96 h [14]. Following this incubation, parasites were washed, resuspended in M199-pH7.4–10% FBS (pre-warmed at 32°C), and then used to infect murine peritoneal macrophages (collected from BALB/c mice) at a parasite to macrophage ratio of 10∶1. Infected cultures were incubated for 4 h at 32°C in a humidified 5% CO_2_/95% air environment, then washed with pre-warmed medium to remove free extracellular parasites, and further incubated for 3 days. Intracellular amastigotes were co-harvested with macrophages at 24 h, 48 h and 72 h post-infection using a protocol that has shown to ‘freeze’ *L. donovani* gene expression levels instantly [7]. Infection rates were monitored using control plates, as described elsewhere [15]; up to 200 cells were counted in order to determine the percentage of infected macrophages and the average number of amastigotes by infected macrophages. The whole experiment was done in 2 biological replicates.

### RNA isolation and cDNA synthesis

RNA sampling protocols, RNA isolation and quality control were performed as described elsewhere [16]. First strand cDNA was synthesized from 150 ng total RNA of promastigote samples and from 1 µg total RNA of mixed amastigote-macrophage samples, using a 18mer oligo(dT) and Transcriptor Reverse Transcriptase, according to the manufacturer's instructions (Roche Applied Science). The resulting cDNA was diluted 10-fold with DEPC-treated water (Ambion) for further use.

### Real-time quantitative PCR

From the cDNA diluted samples, 2 µL was used as template in 25 µL SYBR Green-based quantitative PCR (qPCR) reactions on the iCycler (Bio-Rad), as previously described [16], with the only modification that amplification was done for 34 cycles [6]. We analyzed 12 *Leishmania*-specific genes, with predicted function in transport (*LbAQP1*, *MRPA*), thiol-based redox metabolism (*GSH1*, *GSH2*, *ODC*, *TRYR*), cellular reduction (*ACR2*, *TDR1*), RNA poly(A)-tail metabolism (*PABP*, *PAP14*), cytoskeleton function (*Actin*) and ribosomal function (*S8*). Primer sequences, parameters and reproducibility of the respective qPCR assays have been described previously [6].

### Data analysis and statistics

Analysis was performed on duplicate biological samples that were each assayed in triplicate. The arithmetic average threshold cycle (Ct) was used for data analysis. The Ct values of each qPCR run were imported as Excel files into qBase*Plus* 1.3 (Biogazelle NV, Zulte, Belgium), a software for real-time PCR data analysis based on the geNorm method [17] and qBase technology [18]. Three genes (*ACR2*, *GSH2*, *PAP14*) showed the most stable expression through parasite life stages in our sample panel (geNorm stability mean M-value and mean coefficient of variation lower than 0.45 and 20%, respectively) and data were normalized to their geometric mean. The use of multiple control genes allows more accurate and reliable normalization of gene expression data [17].

The linear component of the variability of the expression level of each gene during *in vitro* growth was modelled independently for each isolate using linear regression. The main predictor was the development time of the parasite during *in vitro* promastigote or intracellular amastigote growth/differentiation. The time point 24 h was considered as the baseline time in the regression models, in order to prevent negative values for gene expression levels, a biological impossibility. The constant terms of the regression models were used as measure of the baseline expression of a given gene, with the criterion of significance that the constant terms differed by at least 2-fold from the lowest constant term in the series.

In addition, the fold change (FC) of gene expression of (i) stationary *versus* logarithmic phase promastigotes (time points 120 h and 168 h *versus* 24 h and 72 h of the growth curves, respectively); (ii) late-differentiating phase amastigotes *versus* early-differentiating phase amastigotes (time points 72 h *versus* 24 h and 48 h post-infection macrophages, respectively); and (iii) stationary phase promastigotes (time points 120 h and 168 h of the growth curves) *versus* early-differentiating phase amastigotes (time points 24 h and 48 h post-infection macrophages) was determined for each parasite isolate. FC were considered significant if they satisfied a 2-fold cutoff and *P*<0.01, as recommended elsewhere [19]. All analyses were performed using the statistical software Stata 10 (StataCorp).

## Results

### Molecular typing of the tested isolates

**Figure 1:**
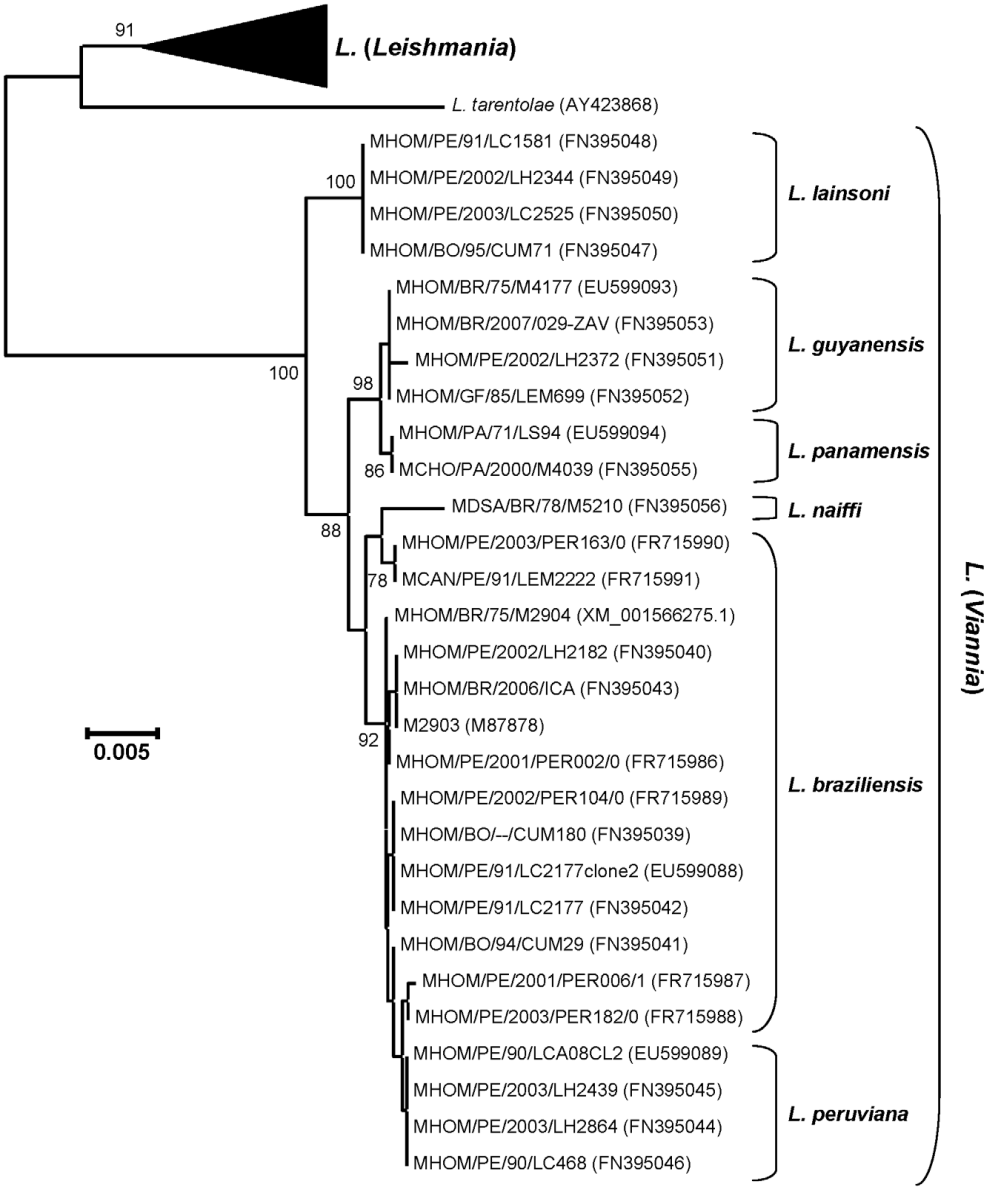
Neighbor-Joining dendrogram based on p-distances of the *hsp*70 sequences determined in this study, aligned with those from Fraga *et al.* [9] as a reference set. The total alignment contains 1380 nucleotides. Bootstrap support of the branches was inferred from 2000 replicates, and is shown in percentages at the internodes when exceeding 70%. The tree is drawn according to the scale on the left, expressed as distance per nucleotide. Different species are depicted on the right, whereby species of the *L.* (*Leishmania*) subgenus are condensed. The tree was rooted on the branch leading to *L.* (*Viannia*). Isolates are indicated with their WHO code wherever possible, with accession numbers between brackets. Accessions starting with XM are derived from a contemporary annotation of full genome sequences, and are GenBank specific.

Out of the 5 isolates, 4 (PER002, -006, -104 and -182) clustered in a well-supported group (bootstrap value of 92%) constituted by several reference strains of the *L. braziliensis* complex (Fig. 1). The fifth isolate, PER163, has 100% sequence identity with the MLEE typed *L. braziliensis* reference strain LEM2222, and they cluster with a bootstrap support of 78%. Even though PER163 has more sequence similarity (99.64%) with the main *L. braziliensis* group than with *L. naiffi* (99.49%), it clusters with the latter without bootstrap support.

### *In vitro* phenotypes

A series of parameters were measured during the growth of promastigotes and the macrophage infection by amastigotes for phenotypic comparison of the 5 isolates. At the promastigote level, our observations led us to the empirical description of 3 main phenotypes (Table 1): (i) slow growth, but high density at stationary phase in PER163 and PER006, (ii) rapid growth and high density at stationary phase in PER104 and PER182, and (iii) slow growth and lower density at stationary phase in PER002. At the amastigote level, 2 main phenotypes were observed (Table 1): (i) high percentage of infected macrophages and high number of amastigotes per macrophage in PER163 and PER104, and (ii) low values for both parameters in PER002, PER006 and PER182.

### Gene expression profiling through *in vitro* life stages

**Figure 2:**
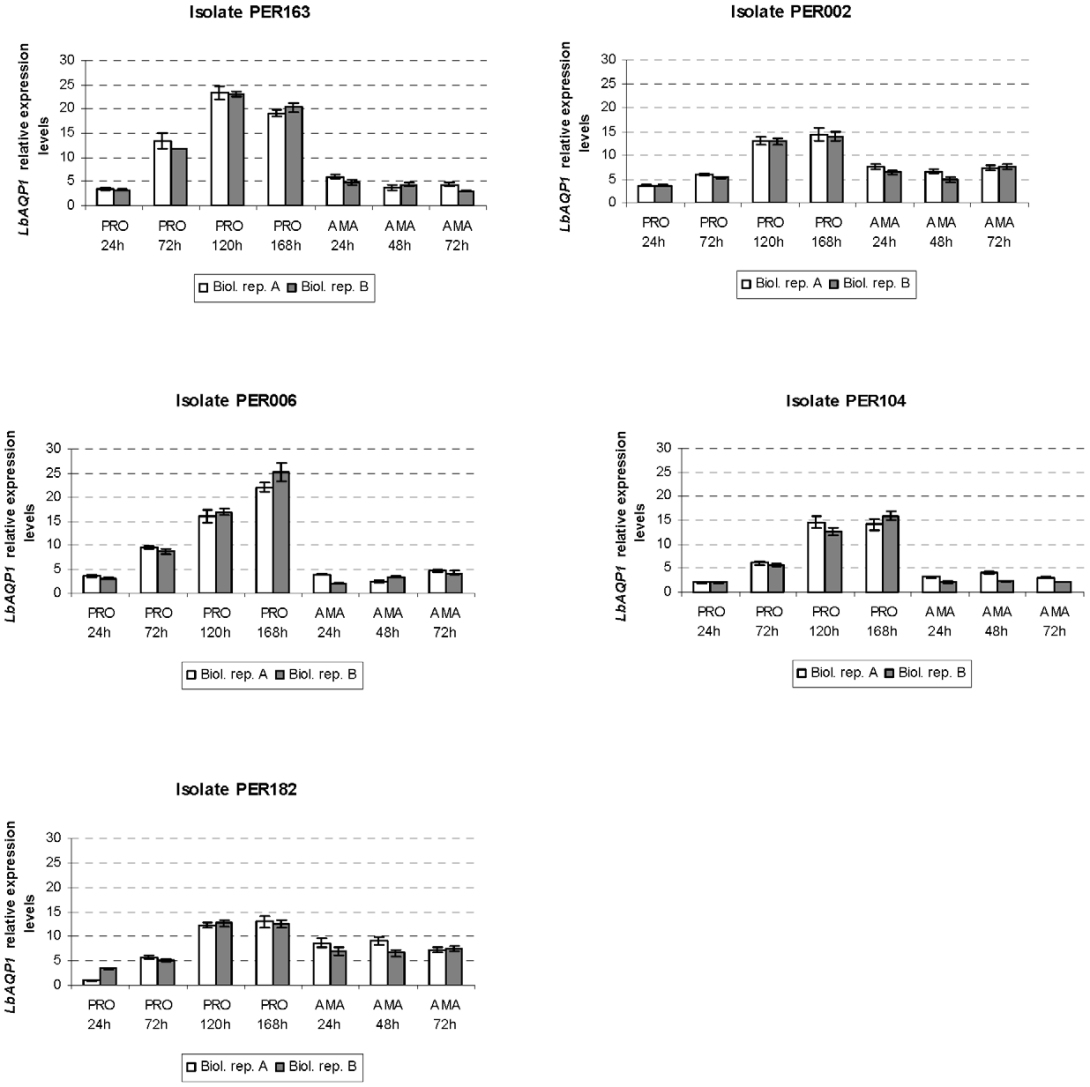
Variation in *LbAQP1* gene expression throughout *in vitro* growth and differentiation of promastigotes and intracellular amastigotes of *L. braziliensis* isolates studied. Time scale in promastigotes (PRO): 24 h, 72 h, 120 h and 168 h of growth. Time scale in intracellular amastigotes (AMA): 24 h, 48 h, and 72 h post-infection macrophages. White and grey bars at each time point represent two experimental biological replicate series (A and B). Normalized expression levels of each gene were rescaled relative to the sample with the lowest expression. Results are expressed as means (± standard errors) of triplicate measurements from one quantitative experiment.

**Figure 3:**
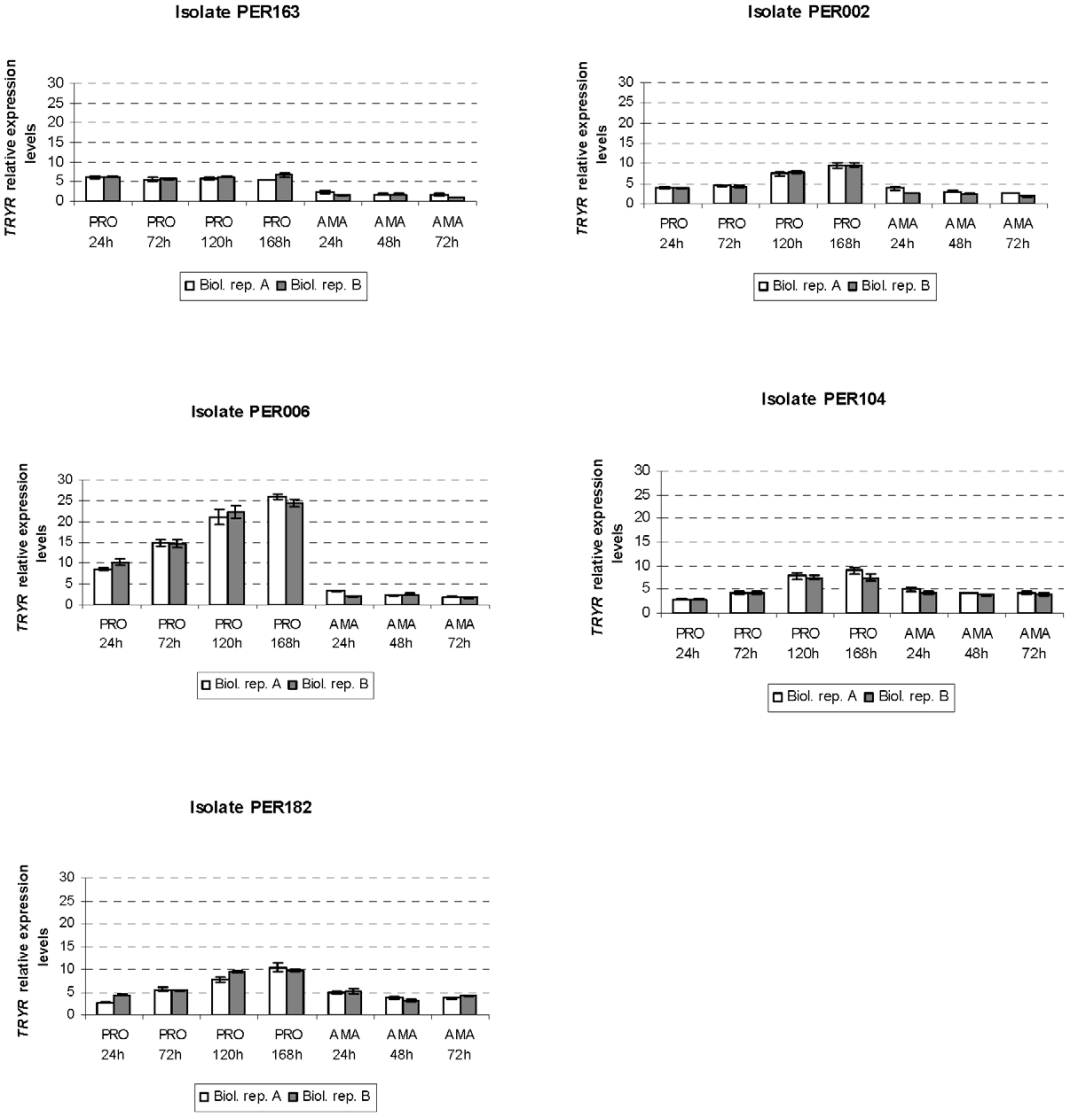
Variation in *TRYR* gene expression throughout *in vitro* growth and differentiation of promastigotes and intracellular amastigotes of *L. braziliensis* isolates studied (see legend of **figure 2**).

**Figure 4:**
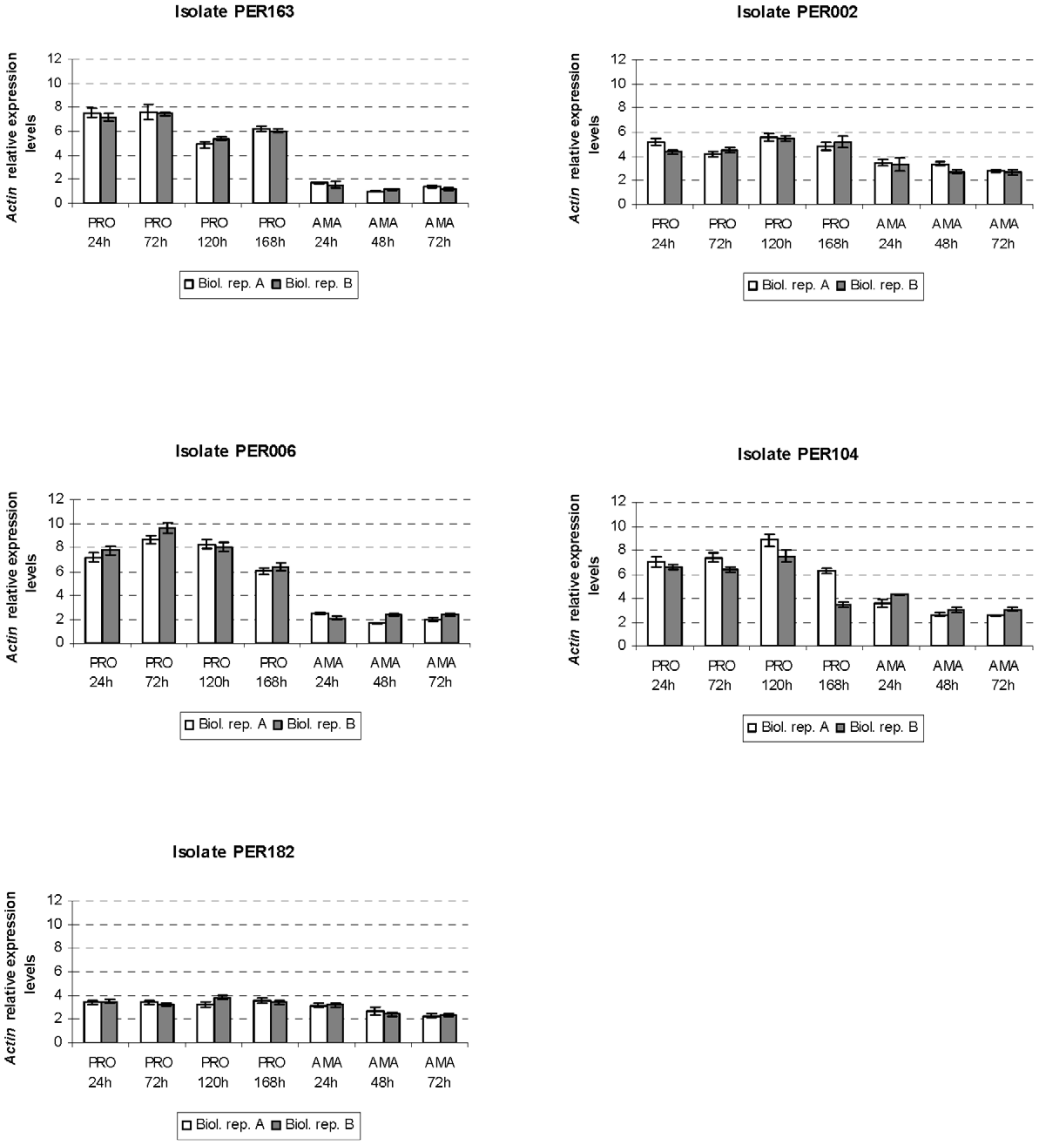
Variation in *Actin* gene expression throughout *in vitro* growth and differentiation of promastigotes and intracellular amastigotes of *L. braziliensis* isolates studied (see legend of **figure 2**).

We obtained highly reproducible gene expression measurements between biological replicates, in both the promastigote and intracellular amastigote developmental time series (Fig. 2, 3, 4), thereby confirming that parasites were manipulated in a standardized way. Visual inspection of the graphs revealed notable differences between life stages in general and between genes according individuals. For instance, *LbAQP1* showed in PER163 and PER006 a clear up-regulation during promastigote development, followed by a 5-fold down-regulation during the transition to amastigotes (Fig. 2). In sharp contrast, *TRYR* showed a similar expression during the 4 time points of promastigote growth curve in PER163, while in PER006 a clear up-regulation was observed (Fig. 3). A last example concerns the expression of *Actin* shown to be down-regulated from promastigote to amastigote in PER163 and PER006, whereas it appeared to be clearly constitutive and at relative low levels in PER182 through the 7 time points analyzed here (Fig. 4).

**Figure 5:**
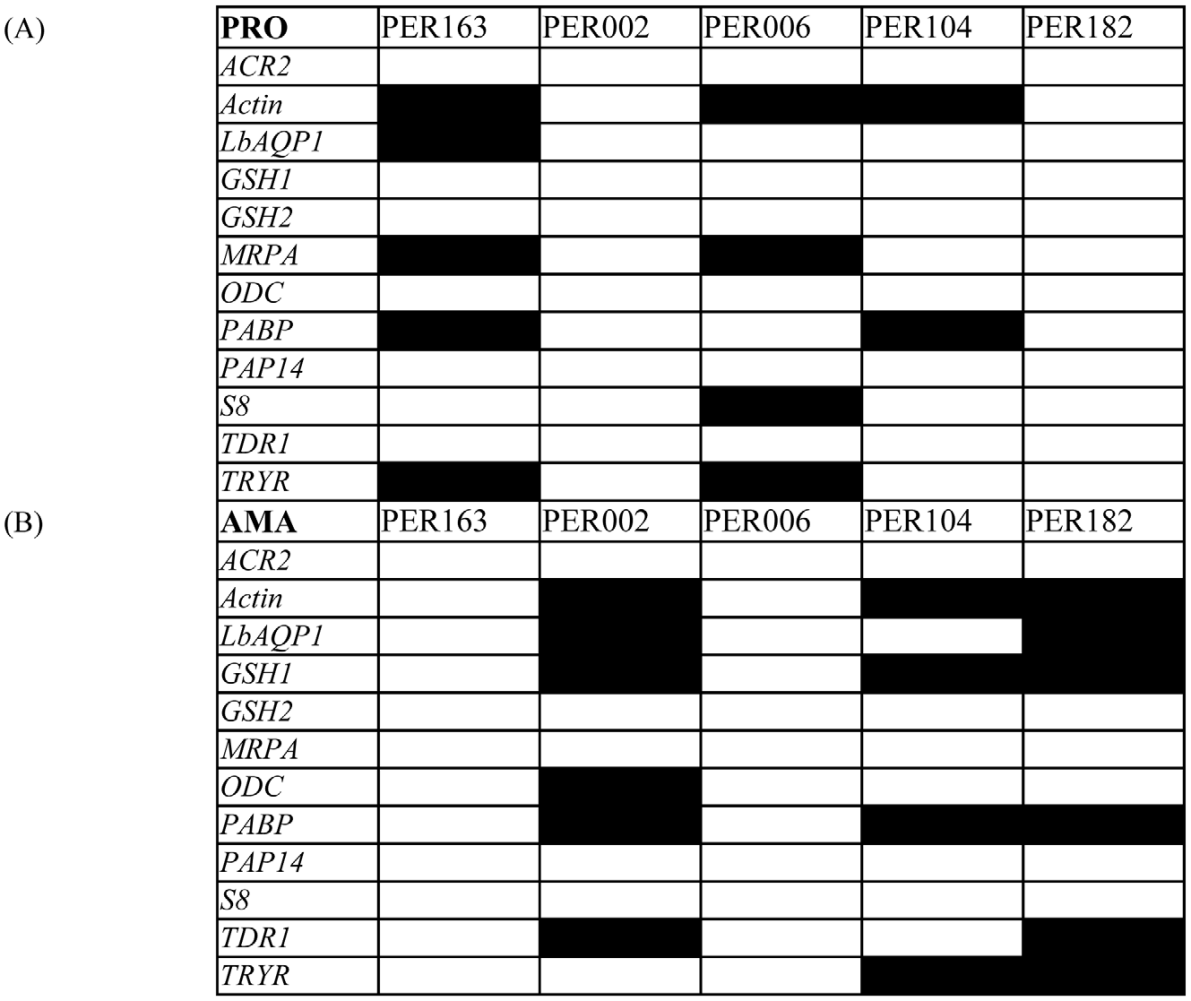
Comparison of baseline expression levels among studied isolates. (A) Gene expression analysis among promastigotes: PRO constant term, measure of the baseline expression of a given gene in the promastigote stage (highlighted in black if significantly higher –by at least 2-fold– than the lowest constant term in the series). (B) Gene expression analysis among amastigotes: AMA constant term, measure of the baseline expression of a given gene in the amastigote stage (highlighted in black if significantly different –by at least 2-fold– from the lowest constant term in the series).

**Figure 6:**
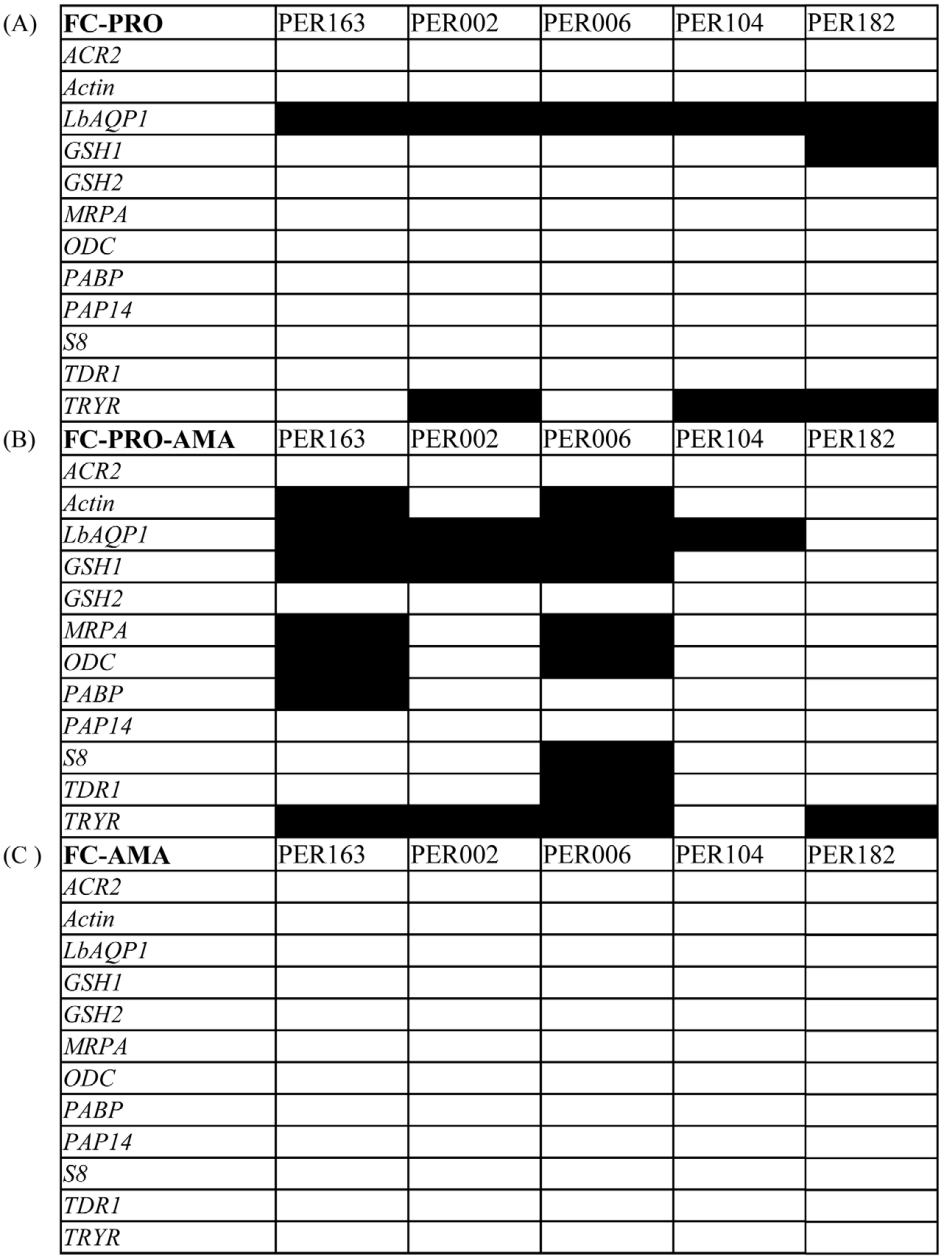
Gene expression modulation in different development stages of studied isolates. Modulation of gene expression can be in the form of increased expression (up-regulation) or decreased expression (down-regulation); a 2-fold cutoff was used here. (A) FC-PRO, measure of gene expression modulation during development of promastigotes (highlighted in black if up-regulation –i.e. at least 2-fold increase– is present). (B) FC-PRO-AMA, measure of gene expression modulation during transition from promastigotes to amastigotes (highlighted in black if modulation, always down-regulation –i.e. at least 2-fold decrease–, is present). (C) FC-AMA, measure of gene expression modulation during development of amastigotes (highlighted in black if modulation is present).

In order to summarize and better visualize the gene expression profiles during the life cycle, we examined 5 parameters in the 5 isolates: the baseline expression levels (given by the constant term of the linear regression models) in (1) promastigotes and (2) amastigotes, and the fold change in mRNA abundance (3) from logarithmic to stationary phase promastigotes (further called FC-PRO), (4) during the transition from stationary phase promastigotes to early-differentiating phase amastigotes (further called FC-PRO-AMA), and (5) from early- to late-differentiating phase intracellular amastigotes (further called FC-AMA). Results are schematized in Figures 5 and 6.

First, when analyzing all parameters together, we identified 3 genes that were constitutively expressed during the whole life cycle and in the 5 isolates: *ACR2*, *GSH2* and *PAP14*. Secondly, analysis of the baseline expression in promastigotes (Fig. 5A) discriminated 2 groups of parasites: (i) PER002 and PER182, which showed the lowest baseline levels for the 12 genes studied here and (ii) PER163, PER006 and PER104 which showed significantly higher baseline levels for 2 to 5 genes (out of the following set: *Actin*, *LbAQP1*, *MRPA*, *PABP*, *S8* and *TRYR*). Thirdly, analysis of the baseline expression in amastigotes (Fig. 5B) also discriminated 2 groups of parasites: (i) PER163 and PER006 showed the lowest baseline for the 12 genes at the amastigote stage, which is in sharp contrast to promastigote data, and (ii) PER002, PER104 and PER182 which showed significantly higher baseline expression of 4 to 6 genes (out of the following set: *Actin*, *LbAQP1*, *GSH1*, *ODC*, *PABP*, *TDR1* and *TRYR*). Fourthly, up-regulation of gene expression during promastigote differentiation (FC-PRO ≥2, Fig. 6A) was observed for up to 3 genes out of the following set: *LbAQP1*, *GSH1* and *TRYR*. As did other parameters, the FC-PRO also discriminated PER163 and PER006 from other isolates, as the former showed the lowest number of up-regulated genes. Fifthly, down-regulation during the promastigote-amastigote transition (FC-PRO-AMA ≥2, Fig. 6B) was observed in all isolates, but to a very different extent: (i) involving 7 and 8 genes in PER163 and PER006 respectively, (ii) 3 genes in PER002 and (iii) one gene only in PER104 and PER182. Sixthly, and in stark contrast to previous parameters, we did not observe up-regulation of any gene in any isolate during amastigote development (FC-AMA <2, Fig. 6C).

## Discussion

Most of the *Leishmania* genome is reported to be expressed constitutively [4], [5], a small fraction of it only (estimated at 5,7% in *L. infantum* and 9% in *L. braziliensis*; [4], [5]) being regulated during the life cycle. In our attempt to explore the diversity of gene expression profiles within a single species -*L. braziliensis* in our case- we thus focused our analysis on a set of genes that were more prone to show variation in gene expression in *L. braziliensis* when assaying promastigotes [6].

Overall, the fine monitoring of the expression of these 12 genes over seven time points of the promastigote and amastigote stages supported previous data on the dynamics of up-/down-regulation during the life cycle. When considering the present sample of isolates as a single pool and looking at the general trend of the expression profiles, regulation appeared to be concentrated around the stationary phases of promastigotes: (i) up-regulation during promastigote growth (3 genes in total), (ii) down-regulation during the promastigote-amastigote transition (9 genes in total) and (iii) no gene showing any up-regulation during the period of monitoring (24 to 72 hours) of amastigote growth. Several reports on the expression profile of the intracellular amastigote stage compared with logarithmic or stationary phase promastigotes or both in several *Leishmania* spp. showed a highly significant predominance of down-regulated over up-regulated genes in amastigotes [4], [20], [21]. Altogether, our results are in agreement with the previously formulated hypothesis that *Leishmania* are pre-adapted for intracellular survival [4], [5].

However, when considering each isolate separately, our results clearly illustrate the unique character of the different parasite populations in terms of gene expression dynamics. Indeed, major differences were observed between the isolates studied here. This concerned essentially the baseline expression levels in promastigotes and amastigotes and the degree of down-regulation during the transition from promastigotes to amastigotes. In terms of similarity of gene expression patterns, 2 groups of parasites could be distinguished: PER163 and PER006 on one hand, and PER002, PER104 and PER182 on the other hand. This grouping was not dependent of the genetic relationships among isolates as inferred from the *Hsp70* sequences (Fig. 1) nor confirmed by other genetic markers (MLMT and AFLP, unpublished results). Strikingly, the isolates PER163 and PER006, which have a higher baseline expression level for several genes in promastigotes than the other isolates and the highest number of down-regulated genes during the transition from promastigotes to amastigotes, were the 2 isolates showing the lowest constitutive levels of baseline expression in amastigotes: the coherence between the different parameters studied here further supports the validity of our methodology. Reciprocally, isolates PER002, PER104 and PER182 call the attention by their low baseline expression levels in promastigotes, the few down-regulated genes during the transition from promastigotes to amastigotes, and the higher level of baseline expression in intracellular amastigotes compared to the isolates PER163 and PER006.

Gene expression diversity in the present sample is not a surprise, considering the phenotype diversity of the analyzed *L. braziliensis* isolates: growth rate at promastigote stage, *in vitro* infectivity for macrophages, *in vitro* susceptibility to Sb^V^ and Sb^III^ or the treatment outcome in patients. The choice of genes likely also played a role in the observed diversity, as they encode proteins with roles in transport, thiol-based redox metabolism, cellular reduction and RNA metabolism, and were previously shown to undergo significant changes in expression in some of the phenotypes present here [7], [22]. However, our aim was not to search any correlation between the gene expression patterns and the main phenotypes observed. This would require another experimental set-up, including (i) a larger sample size, (ii) a broader coverage of the transcriptome and (iii) complementary studies at other ‘omic levels. Proteomic approaches are very relevant, considering the importance of post-transcriptional regulation in Trypanosomatids [23]. But, even more important, the metabolome should be explored, because of its closest position to the phenotype [24].

In conclusion, our results show that the gene expression pattern of a strain is not necessarily representative of a given species. They remind us of an essential feature of natural populations of *Leishmania*: diversity. We recommend to keep this dimension in mind in any future work and to take extreme care when comparing the profiles of different species represented by single strains and extrapolating functional differences between them.

### Accession Numbers

The accession numbers for the genes analyzed in this study are as annotated at *L. braziliensis* GeneDB database (http://www.genedb.org/Homepage/Lbraziliensis). *LbAQP1* (GeneDB ID LbrM.31.0020) encodes an aquaglyceroporin. *MRPA* (LbrM.23.0280) codes for an ABC-thiol transporter. *GSH1* (LbrM.18.1700) encodes a putative gamma-glutamylcysteine synthetase (γ-GCS). *GSH2* (LbrM.14.0880) codes for a putative glutathione synthetase (GS). *ODC* (LbrM.12.0300) codes for a putative ornithine decarboxylase. *TRYR* (LbrM.05.0350) encodes trypanothione reductase. *ACR2* (LbrM.32.2980) encodes a putative Sb^V^/As^V^ reductase according to the orthologous *ACR2* sequence in *L. major* (GeneDB ID LmjF.32.2740; GenBank accession number AY567836.1). *TDR1* (LbrM.31.0550) encodes a thiol-dependent reductase 1. *PABP* (LbrM.30.2560) encodes a putative RNA-binding protein. *PAP14* (LbrM.14.1350) codes for a putative poly(A) polymerase. *Actin* (LbrM.04.1250) codes for the actin protein. *S8* (LbrM.24.2160) encodes a putative 40S ribosomal protein S8.

## References

[1] Barak E, Amin-Spector S, Gerliak E, Goyard S, Holland N (2005). Differentiation of *Leishmania donovani* in
host-free system: analysis of signal perception and
response.. Mol Biochem Parasitol.

[2] Haile S, Papadopoulou B (2007). Developmental regulation of gene expression in trypanosomatid
parasitic protozoa.. Curr Opin Microbiol.

[3] Saxena A, Lahav T, Holland N, Aggarwal G, Anupama A (2007). Analysis of the *Leishmania donovani*
transcriptome reveals an ordered progression of transient and permanent
changes in gene expression during differentiation.. Mol Biochem Parasitol.

[4] Alcolea PJ, Alonso A, Gómez MJ, Moreno I, Domínguez M (2010). Transcriptomics throughout the life cycle of *Leishmania
infantum*: high down-regulation rate in the amastigote
stage.. Int J Parasitol.

[5] Depledge DP, Evans KJ, Ivens AC, Aziz N, Maroof A (2009). Comparative expression profiling of *Leishmania*:
modulation in gene expression between species and in different host genetic
backgrounds.. PLoS Negl Trop Dis.

[6] Adaui V, Schnorbusch K, Zimic M, Gutiérrez A, Decuypere S (2011). Comparison of gene expression patterns among *Leishmania
braziliensis* clinical isolates showing a different *in
vitro* susceptibility to pentavalent antimony.. Parasitology.

[7] Decuypere S, Vanaerschot M, Rijal S, Yardley V, Maes L (2008). Gene expression profiling of *Leishmania (Leishmania)
donovani*: overcoming technical variation and exploiting
biological variation.. Parasitology.

[8] Yardley V, Ortuño N, Llanos-Cuentas A, Chappuis F, De Doncker S (2006). American tegumentary leishmaniasis: Is antimonial treatment
outcome related to parasite drug susceptibility?. J Infect Dis.

[9] Fraga J, Montalvo AM, De Doncker S, Dujardin JC, Van der Auwera G (2010). Phylogeny of *Leishmania* species based on the
heat-shock protein 70 gene.. Infect Genet Evol.

[10] Garcia L, Kindt A, Bermudez H, Llanos-Cuentas A, De Doncker S (2004). Culture-independent species typing of neotropical
*Leishmania* for clinical validation of a PCR-based assay
targeting heat shock protein 70 genes.. J Clin Microbiol.

[11] da Silva LA, de Sousa Cdos S, da Graça GC, Porrozzi R, Cupolillo E (2010). Sequence analysis and PCR-RFLP profiling of the hsp70 gene as a
valuable tool for identifying *Leishmania* species associated
with human leishmaniasis in Brazil.. Infect Genet Evol.

[12] Montalvo AM, Fraga J, Monzote L, Montano I, De Doncker S (2010). Heat-shock protein 70 PCR-RFLP: a universal simple tool for
*Leishmania* species discrimination in the New and Old
World.. Parasitology.

[13] Puentes F, Diaz D, Hoya RD, Gutíerrez JA, Lozano JM (2000). Cultivation and characterization of stable *Leishmania
guyanensis* complex axenic amastigotes derived from infected
U937 cells.. Am J Trop Med Hyg.

[14] Teixeira MC, de Jesus Santos R, Sampaio RB, Pontes-de-Carvalho L, dos-Santos WL (2002). A simple and reproducible method to obtain large numbers of
axenic amastigotes of different *Leishmania*
species.. Parasitol Res.

[15] Gamboa D, Torres K, De Doncker S, Zimic M, Arevalo J (2008). Evaluation of an *in vitro* and *in
vivo* model for experimental infection with *Leishmania
(Viannia) braziliensis* and *L. (V.)
peruviana*.. Parasitology.

[16] Decuypere S, Rijal S, Yardley V, De Doncker S, Laurent T (2005). Gene expression analysis of the mechanism of natural Sb(V)
resistance in *Leishmania donovani* isolates from
Nepal.. Antimicrob Agents Chemother.

[17] Vandesompele J, De Preter K, Pattyn F, Poppe B, Van Roy N (2002). Accurate normalization of real-time quantitative RT-PCR data by
geometric averaging of multiple internal control genes.. Genome Biol.

[18] Hellemans J, Mortier G, De Paepe A, Speleman F, Vandesompele J (2007). qBase relative quantification framework and software for
management and automated analysis of real-time quantitative PCR
data.. Genome Biol.

[19] McCarthy DJ, Smyth GK (2009). Testing significance relative to a fold-change threshold is a
TREAT.. Bioinformatics.

[20] Holzer TR, McMaster WR, Forney JD (2006). Expression profiling by whole-genome interspecies microarray
hybridization reveals differential gene expression in procyclic
promastigotes, lesion-derived amastigotes, and axenic amastigotes in
*Leishmania mexicana*.. Mol Biochem Parasitol.

[21] Rochette A, Raymond F, Corbeil J, Ouellette M, Papadopoulou B (2009). Whole-genome comparative RNA expression profiling of axenic and
intracellular amastigote forms of *Leishmania
infantum*.. Mol Biochem Parasitol.

[22] Ashutosh, Sundar S, Goyal N (2007). Molecular mechanisms of antimony resistance in
*Leishmania*.. J Med Microbiol.

[23] Rochette A, Raymond F, Ubeda JM, Smith M, Messier N (2008). Genome-wide gene expression profiling analysis of
*Leishmania major* and *Leishmania
infantum* developmental stages reveals substantial differences
between the two species.. BMC Genomics.

[24] t'Kindt R, Scheltema RA, Jankevics A, Brunker K, Rijal S (2010). Metabolomics to unveil and understand phenotypic diversity
between pathogen populations.. PLoS Negl Trop Dis.

